# Dataset for historical lighthouses and light aids to navigation (LAN). England and Wales, 1514–1911.

**DOI:** 10.1016/j.dib.2020.105991

**Published:** 2020-07-07

**Authors:** Oliver Buxton Dunn, Eduard J. Alvarez-Palau

**Affiliations:** a University of Cambridge, UK; b Universitat Oberta de Catalunya, Spain

**Keywords:** Lighthouses, Coastal navigation, Transport infrastructure, Historical geography

## Abstract

We describe ‘LAN’, a geospatial data set that documents the development of coastal lights that guided ships around England and Wales from medieval times to 1911. As an additional benefit, LAN provides visibility range of individual lights from 1831. The authors collected all data from scholarly literature and historical coastal navigational charts and official lighthouse surveys. The sources were selected for their good coverage of coastal lighting. LAN allows users to track the development of coastal lighting over time by presenting snapshots of this service as it was during several benchmark periods.

Specifications Table

**Table:** 

**Subject**	Human Geography
**Specific subject area**	Economic History, Transport Infrastructure, Historical Geography, Maritime History
**Type of data**	Shapefile Table
**How data were acquired**	We acquired the data from archived records and published secondary sources. The authors filmed the sources in public archives and transcribed the data from the imagery later.
**Data format**	Raw
**Parameters for data collection**	A primary consideration for the data collection was to maximise geographical and temporal coverage. Another was source reliability and detail. The sources give data for Ireland and Scotland and we plan to add this data to LAN at a later stage.
**Description of data collection**	The data was collected using Optical Character Recognition tools (OCR), GIS annotation, and manual data-entry methods. Individual lighthouse locations were copied from admiralty lists and charts published in official journals from 1832, 1852 and 1912. Coastal navigation charts showed locations for lights in the 1693 and 1753 benchmarks. A published archaeological survey gave the medieval light locations. We compared the charts with modern geographical information systems to get coordinate positions for some lights.
**Data source location**	England and Wales
**Data accessibility**	Repository name: UK Data Service Reshare Data identification number: 854,172 Direct URL to data: 10.5255/UKDA-SN-854,172

## Value of the data

• LAN shows growth in numbers of light aids to navigation and light coverage at sea over a 400-year period around England and Wales.
• Historians and social scientists alike can benefit from the data, which illuminate the development of coastal lighting and the extension of its coverage at sea over a long historical period.
• The data can cast light on the development of coastal infrastructure, the maritime economy, safety at sea, and transport costs.
• LAN is designed for use with geographical information systems (GIS).

## Data description

**Fig. 1:**
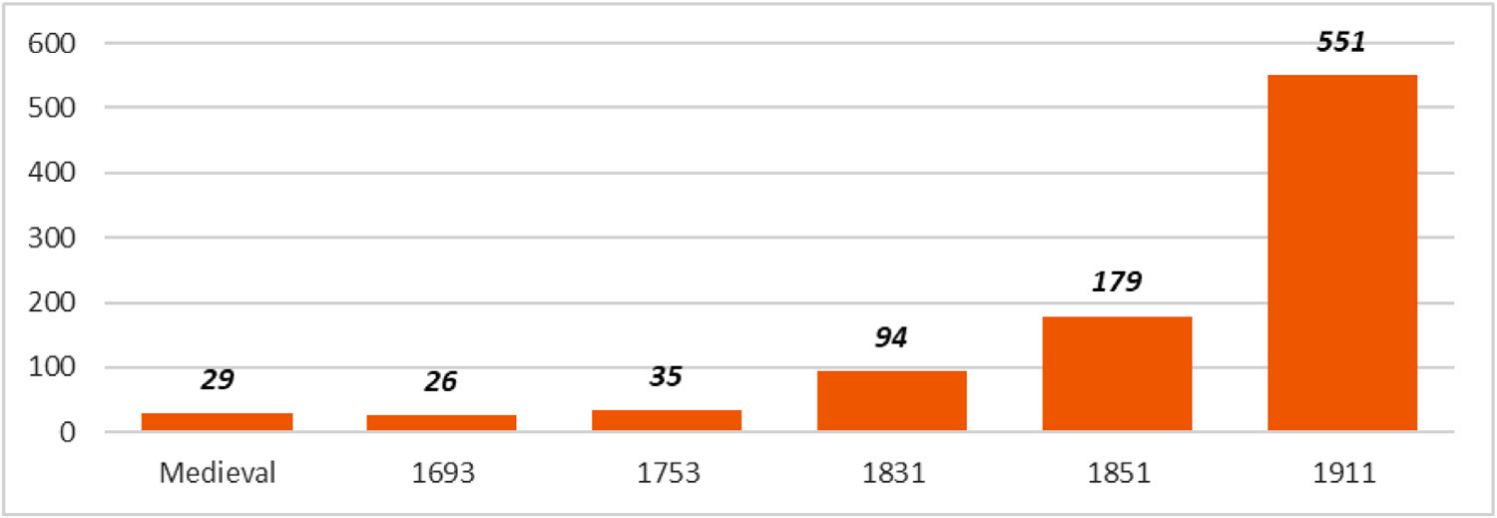
Number of lights (all types) reported by benchmark period in LAN. (Establishments fitted with multiple lights are counted as one observation.).

**Fig. 2:**
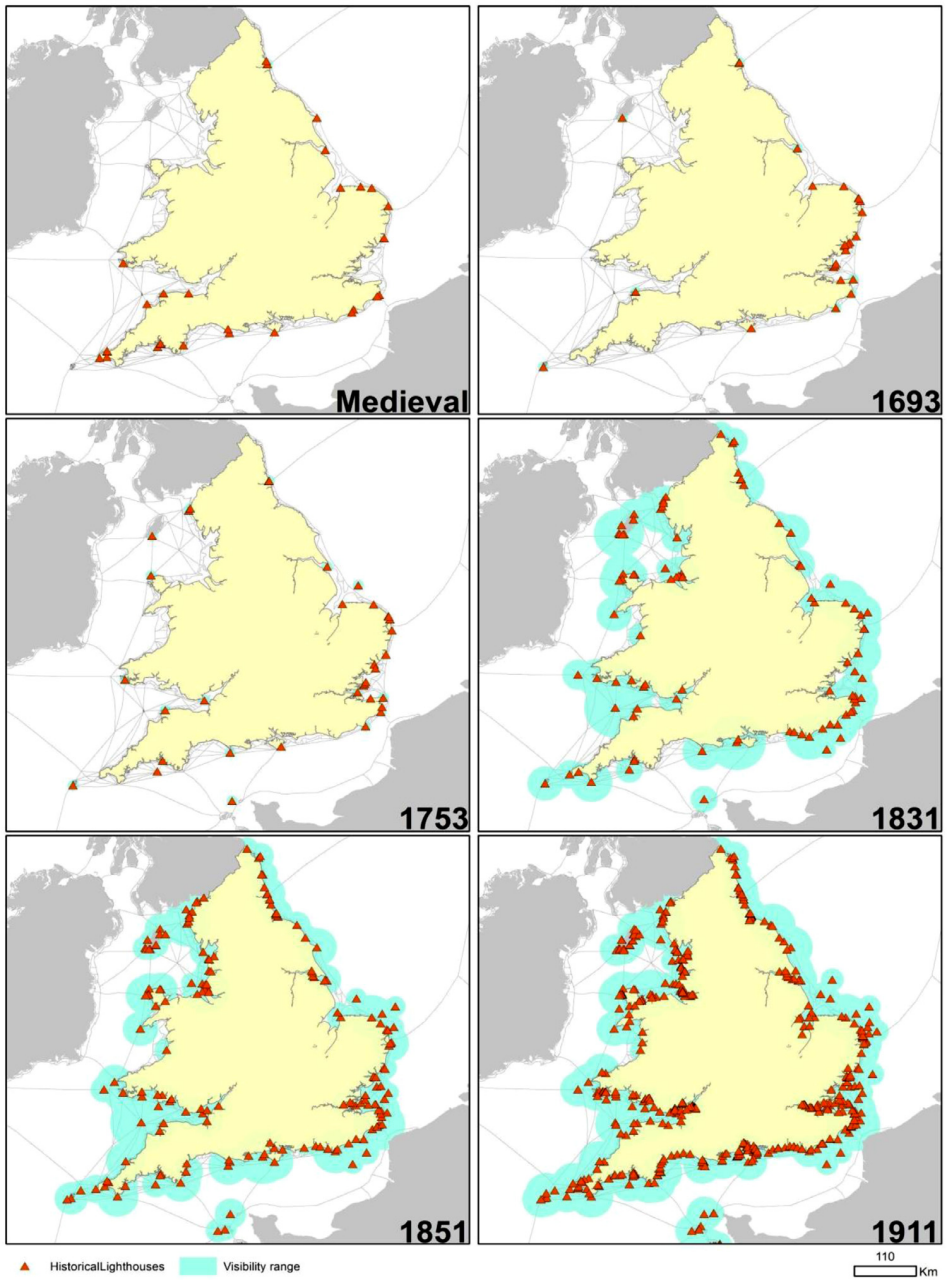
Evolution of lighthouses and visibility ranges over time. Grey lines show coastal routes and port connections [1]. If the sources gave no visibility value (1753 and earlier) we take the average values from Naish [2].

Fig. 1 summarises by benchmark period the number of light establishments of all kinds included in LAN. Table 1 lists LAN variables. This is followed by a description in the next section of the methods used to generate each variable type. We grouped variables by benchmark period. Fig. 2 maps the data set in ArcGIS. It shows how the number of light aids to navigation increased over time and where they were built in different benchmarks. It also shows the evolution of light coverage using our own estimates for benchmark periods before 1831, and actual light coverage data collected from the sources after this date.

**Table 1:** LAN variables types.

Field	Description
*FID*	Unique ID for each row in the table
*Shape*	Point for the location of the lighthouse
*Name*	Unique name of each lighthouse
*LH_Med*	Number of lights established on site in medieval period (0 = no lighthouse establishment)
*LH_1693*	Number of lights in 1693 (0 = no lighthouse establishment)
*LH_1753*	Number of lights established on site 1753 (0 = no lighthouse establishment)
*LH_1831*	Number of lights established on site in 1831 (0 = no lighthouse establishment)
*Range_1831*	Night-time visibility range of lights in 1831 (nautical miles)
*LH_1851*	Number of lights established on site in 1851 (0 = no lighthouse establishment)
*Range_1851*	Night-time visibility range of lights identified from our sources for 1851 (nautical miles)
*LH_1911*	Number of lights established on site in 1911 (0 = no lighthouse establishment)
*Range_1911*	Night-time visibility range of lights identified from our sources for 1911 (nautical miles)
*Height*	Height of lighthouse lantern above sea level (feet) (1831)
*YearBuilt*	First recorded year of construction
*Point_X*	Geographical coordinate X of the lighthouse
*Point_Y*	Geographical coordinate Y of the lighthouse

## Experimental design, materials, and methods

LAN draws on sources originally created by historical maritime authorities, or historians, that detail navigational lights and their attributes as they were in the benchmark years. Sources were consulted at the British Library, Cambridge University Library, and the UK Hydrographic Office Archive, Taunton.

LAN is divided into six benchmark years, which follow the original publication dates of the sources used (1693, 1753, 1832, 1852, and 1912). The Medieval benchmark data was taken from secondary sources. Dates of origin of medieval lights are rarely available, so a broad periodization was employed. Other benchmark periods represent lighting in the year prior to the publication date.

LAN tracks *active* lights with a numeric code as they emerge or disappear from the historical sources consulted. If lights were listed or depicted as active in a source of information for a given period, they are given a value from one to six. This number shows lights that existed at the time, and the number of lights displayed from the lighthouses, leading-lights, lightships, light beacons and harbour lights LAN includes. Note that multiple lights were displayed from all kinds of light aids to navigation. A light's absence in any period is represented by a zero. Absence arises in LAN if lights had not yet been built, or if they had been deactivated at some point after construction. Therefore, LAN shows active lights in different periods only. A zero is given in all other cases.

The resulting shapefile is ready for use with GIS. We also provide a table. The variables in both the shapefile and table are the same. Individual light observations are arranged by row; the different variables are given in columns, all separated by benchmark periods.

Cross-benchmark variables:

*FID* is the ID we gave to each light observation.

*Shape* is the geometric type of data for each light observation: in this case, a point.

The *Name* variable is the light name in standardised form. Standardisation was necessary to show continuity of light existence at a given location over time. Light names changed at locations over this period, even when there was a continuous presence of lights as shown in LAN.

The naming and description of lights in the sources were sometimes ambiguous. This was especially the case with smaller harbour lights listed in 1911, e.g. ‘Southampton Town Pier’ (Southampton Town Quay), or the ‘Liverpool Georges’ (Liverpool George's Dock).

Light locations were found using online sources, Google Earth Pro, scholarly literature, and other historical maps that give fine-level detail. Once located, we generated the variables *Point_X* and *Point_Y*.

*Height* variable data were collected from the hydrographic office *Light-List* of 1832 and cover lights as they were in 1831–32.

*YearBuilt* gives the construction date of lights in named locations given by the hydrographic Office *Lists*. The year lights were first erected were collected from the light-list published in 1832. The original variable title was ’erection date’.

We will now describe data collection methods and the sources, starting with the Medieval benchmark, and ending with 1911.

*Medieval* benchmark-specific variables:

Lights in this benchmark possess fewer attributes compared with other lights in the data set because of a general lack of evidence dating from the period.

Hague and Christie [3] provided a map that indicates which lighthouses existed in the medieval period based on their historical and archaeological research. We took our information from their medieval map of lights in England and Wales. Medieval lights were small in scale and mostly candle or oil powered. Some, such as Dover, were Roman in origin.

*LH_Med* is the number of lights shone from individual light establishments. We generally assume a value of 1.

The available sources do not give range data for the medieval benchmark period. Therefore, we did not include a *Range* variable in the medieval benchmark. Naish [2] estimates visibility range between 1.6 and 5.3 standard miles respectively if candles or coal fires were used. We suggest his estimates can be interpolated to add visibility range in these cases.

*1693 and 1753* benchmark variables:

Two published editions of Collins’ *Great-Britain's Coastal Pilot* [4,5] showed where lights existed in 1693 and 1753. We consulted both editions at the Cambridge University Library. Collins conducted his surveys rigorously. Whilst his cartographic surveys are likely to be imperfect because of limits to contemporary surveying technology, we assume his observations of lights are reasonably accurate.

Collins depicted lighthouses on his charts alongside smaller light aids to navigation, such as fire beacons. We annotated these using satellite imagery in Google Earth Pro. This approach provided modern geographical decimal coordinates that determine the location of the light.

The number of lights per establishment is given by *LH_1693* and *LH_1753*. Zero indicates there was no light in this period. Some light establishments possessed more than one light or even multiple towers, e.g. leading lights that when seen in alignment indicated ship position at sea. Single towers also used light clusters to aid their identification by navigators.

Collins’ charts indicate active lights using depictions of fire and smoke. Deactivated lights were indicated by the omission of these symbols. There was a risk we could misinterpret Collins’ depictions. To guard against this, we confirmed cases of suspected extinguished lights in Hague and Christie [3]. Cases of extinguishment were few and are now well-documented, meaning we were able to find confirmatory information for these examples reliably. Deactivated lights are given a zero value.

Collins did not provide clear information about light range, so we did not include a range variable for these benchmarks. One could, as with the ‘medieval’ lights, consider interpolating Naish's [2] range estimates in the data.

*1831, 1851 and 1911* benchmark-specific variables:

The UK Hydrographic Office (hereafter, UKHO) published *Lists of Lights* annually from 1832 and were used to create the 1831, 1851, and 1911 benchmark data [6], [7], [8]. This was a journal originally sold to provide up-to-date information about British (including Ireland) harbour lights, lighthouses, and light-vessels. UKHO *Lists* detailed all active lights in printed tables.

UKHO *Lists* indicate locations of all *active* lights and other useful information attributable to them for the year prior to publication. Some lights were delisted between editions by UKHO, and where this was the case a zero value is given for the light observation in LAN because we assume these lights were deactivated. We do not record information about why lights were deactivated.

UKHO provided geographical coordinates, which were useful for locating some lights. Some original coordinate data, however, proved to be inaccurate when it was transcribed and loaded in modern GIS. We followed UKHO's written descriptions of lights and created new coordinates in cases where the original coordinates were incorrect. For most observations of lights found in the UKHO Lists, coordinate data was collected with the aid of original UKHO coastal navigation charts that we consulted in the Cambridge University Library and the Hydrographic Office Archive, Taunton. We used charts dated within 5 years of the *List* publication date to ensure the two sources were comparable. UKHO clearly depicted lights on its charts, like Collins, because they were prominent navigational aids. Using all these methods, the authors annotated light locations in an accurate and complete manner.

*LH_1831, LH_1851* and *LH_1911* give the number of lights shone from individual light establishments*.*

UKHO *Lists* provided *Range_1831, Range_1851* and *Range_1911.* The original description in these sources for the light range variable was in all *Lists* given as ‘Distance in miles (nautical miles) at which they are easily seen in clear weather’.

## Declaration of Competing Interest

None.
